# Editors’ Book Table

**Published:** 1873-11

**Authors:** 


### Editors' Book Table.

[NOTE. — All works reviewed in the columns of the CHICAGO MEDICAL JOURNAL may be found in the extensive stock of W. B. KEEN, COOKE & Co., whose catalogue of Medical Books will be sent to any address upon request.]

BOOKS RECEIVED.

*A Manual of Medical Jurisprudence.* By ALFRED SWAINE TAYLOR, M.D., F.R.S., Fellow of the Royal College of Physicians, and Professor of Medical Jurisprudence and Chemistry, in Guy's Hospital. Seventh American Edition, etc., by JOHN J. REESE, M.D., Professor of Medical Jurisprudence and Toxicology in the University of Pennsylvania, etc., etc. Philadelphia: Henry C. Lea. 1873.

It is no more than strict justice to term this the most useful manual of Medical Jurisprudence within the reach of the majority of American physicians, in saying which we are only expressing verbally, what is practically apparent in the fact, that this is the seventh American edition of the work. Those of our readers who have not yet supplied themselves with this indispensable addition to the physician's library, and whose professional cares do not permit the cultivation of medical jurisprudence as a special study, but must confine themselves to a knowledge of its outlines, will find in the manual before us a most comprehensive summary of the science. The American Editor has enriched the work by the interpolation of copious notes of his own, and also of the former Editors, Dr. Hartshorne and Mr. Penrose.

Of the many portions of the work deserving attention we specially designate the second chapter, referring to the duties and responsibilities and to the unsatisfactory position of medical witnesses, which may be studied with profit by the majority of medical men who are occasionally subjected to the ordeal of the witness stand in a court, and it is certain that a thorough appreciation of the rules herein set forth would go far to enable medical witnesses to protect themselves from the imputations of unfairness so frequently cast upon them.

According to the English code, there are no "medical secrets" recognized by the law, "the law concedes no special privilege of this nature to members of the medical profession." "All questions must be answered, provided they are relevant to the case," which may be a very good law, but is certainly very poor justice, in its application sapping the very foundations of professional confidence. It is gratifying to know that in this country this disagreeable necessity has been removed by a more liberal system of jurisprudence, which declares that "no person duly authorized to practice medicine or surgery shall be allowed to disclose any information which he may have acquired in a professional character, and which information was necessary to enable him to prescribe for such patient as a physician, or to do any act for him as a surgeon." The "rules for the delivery of evidence" are excellent.

The portion of the work devoted to poisons is voluminous and comprehensive. The deduction from the celebrated Wharton case which occurred so recently in this country, is *ex parte*, and will find few endorsers amongst those most familiar with the facts.

The chapters upon the subject of rape are most appropriately terminated by the famous decision of Sancho Panza, the Governor of Barataria, which will cover most of the cases of this sort.

The subject of insanity is well treated; the section relating to the discharge of lunatics is especially commendable, and one sentence merits the earnest attention of jurists upon this side of the water. "One who has been guilty of a heinous crime like murder, should never on any pretense be discharged."

It would be of inestimable value to the cause of justice, and to the medical profession, if our law makers could be clearly impressed with the spirit of the subjoined quotation. "One source of difficulty on these occasions is, that medical witnesses are allowed to be separately sought out and summoned by those who are for and against the commission, and the opinions given by them often exactly neutralize each other. Under these circumstances they are converted into partisans in the cause as much as if they were counsel. It has been well remarked, that a man even unknown to himself, with the purest intentions and the most perfect rectitude, will insensibly lean to the side on which he has been consulted or employed. The public are apt to infer from such conflicting opinions emanating from men of equal experience, that the difference cannot depend essentially upon the medical facts of a case, and that the question might as well or even better be determined by non-professional persons. One remedy for this serious evil would be, that medical experts on such occasions should be selected and appointed by the Lord Chancellor," etc., etc.

Pursuing the same subject (of insanity) the author refers to the apparently irreconcileable opinions of jurists and physicians regarding the legal responsibility of criminal lunatics, and insists, very justly, upon the fallacy of the knowledge of right and wrong as a test of criminal responsibility.

The "relations of medical men with insurance offices" are carefully and justly considered. This subject is now receiving special attention by some of the more prominent medical journals, and it is gratifying to perceive the unanimity of opinion and sentiment upon this subject upon both sides of the water.

It is impossible in the limited space at our command to extend our notice of this valuable manual, which reflects honor upon author, editor, and publisher. H.

*Lectures on Clinical Medicine.* By A. TROUSSEAU, late Professor of Clinical Medicine in the Faculty of Medicine, Paris; Physician to the Hotel Dieu, etc., etc. Translated from the third revised and enlarged edition, by SIR JNO. ROSE CORMACK, M.D., F.R.S.E., Fellow of the Royal College of Physicians of Edinburgh, etc.; and VICTOR BAZIRE, M.D., assistant Physician to the National Hospital for the Paralyzed. Complete in two volumes. Vol I. Philadelphia: Lindsay & Blakiston. 1873.

It would be entirely out of place to attempt a criticism of the work of this great master in medicine, Trousseau; we will, however, take occasion to congratulate the profession upon being placed in possession of this rich store of medical literature in a form at once complete, compact, and cheap. Until the year 1867, Trousseau was unknown except by name to the great mass of American practitioners, only a few of those familiar with his native tongue having had access to his writings. In that year the English edition of five volumes was presented to the profession on this side of the ocean, and, notwithstanding its costliness, met with a most cordial reception. The publishers of that edition have added another to the long list of obligations already due them from American medical students, in bringing the work within the reach of a larger number by reducing its price one-half, and its bulk from five volumes to two, without, however, any essential changes in the subject matter.

While we deprecate blind obedience to prescribed authority, to the exclusion of physiological analysis and logical induction, yet the young practitioner will find no safer guide, the elder no wiser counsellor, than this greatest of modern French physicians, Trousseau. H.

*A Manual of Practical Hygiene*, intended especially for Medical Officers of the Army and for Civil Medical Officers of Health. By EDMUND PARKES, M.D., F.R.S., etc., etc. Fourth edition. Philadelphia: Lindsay & Blakiston. 1873.

The present edition of Dr. Parkes' valuable Manual presents a marked improvement over its predecessor. Originally intended as a text book of hygiene for the use of the students of the Army Medical School, it has been subsequently amplified and made to include subjects of interest not only to practitioners in civil life, but to the laity likewise, who cannot fail to derive much valuable practical instruction from the study of its pages.

The present edition has been advantageously divided into two books. The first comprises the general hygienic relations of water, air, food and drink, soil, dwellings, disposal of sewage, exercise, clothing, etc., etc. The subjects are treated elaborately, and in a manner fully up to the times. While studying a work of this character, one cannot but be astonished at the vast distance attained by hygienic science in advance of its practical application, and feel convinced that, by far the greater portion of the diseases which afflict humanity might be obviated by an intelligent observance of hygienic rules.

The second book comprises the hygenic relations of the soldier, and of course is not so directly valuable to the medical practitioner in civil life, nevertheless there is much even in this portion of the work which is applicable to other social conditions than those for which it was especially written.

We commend the book most heartily to all needing instruction, (and who does not?) in hygiene. To the layman it will be found instructive, and to the physician, who is, *ex officio*, looked up to as instructor in all knowledge having for its object the protection and preservation of human health, and who must therefore keep up with the times, it will be found indispensable: but most of all do we urge upon municipal sanitary bodies the adoption of the work as a text book, for the instruction of officers of health, feeling confident that much good would result from the practical application of hygienic principles to the construction of sanitary codes, which are now in too many instances based upon the crude notions of sciolists and petty politicians.

It is only by means of municipal interposition that communities can be educated in hygiene. Individual instruction by physicians, it is true, will accomplish something, but that amount, even, of instruction cannot be given by those who are themselves uneducated. H.

*Chemistry, Inorganic and Organic, with Experiments.* By CHARLES LOUDON BLOXAM, Professor of Chemistry in King's College, London, etc., etc., etc. From the second and revised English edition. Philadelphia: Henry C. Lea. 1873.

One of the best text books of chemistry yet published, comprising, in three divisions, the chemistry of non-metallic elements and their compounds, the chemistry of metals and metallic compounds, and organic chemistry.

The author has adopted in this edition the atomic system of notation, which of course necessitated the re-arrangement of portions of the work, which, while it may somewhat disturb the accepted ideas of elementary relations, cannot fail ultimately to conduce to certainty in method of study and to accuracy of results.

The study of organic chemistry is too much neglected by medical men, and the ignorance resulting therefrom retards materially our progress in physiological investigation with which chemistry should go hand in hand. The author has devoted a few pages to the consideration of the application of chemical principles to certain of the arts.

The work is well and freely illustrated, and as a whole cannot fail to prove one of the valuable text books in the science, and not less so as a work of reference which should find a place upon every physician's study table. H.

*Report of the Columbia Hospital for Women, and Lying-in Asylum, Washington, D. C.* By J. HARRY THOMPSON, A.M., M.D., Surgeon-in-Chief. With an Appendix. Washington: Government Printing Office. 1873.

In February, 1866, the Columbia Hospital for Women was opened under the auspices of the United States Government in the city of Washington, in anticipation of a charter which was granted by Congress on the following first of June. The Report before us is a *resume* of the operations of the institution from its commencement to the end of June, 1872. It was prepared by the Surgeon-in-Chief, Dr. J. Harry Thompson, who, notwithstanding the brief period of time allotted to the work (about six months) has managed, with the aid of the assistant surgeons in charge of the dispensary attached to the hospital, to present us with a book of four hundred and thirty quarto pages.

The number of surgical operations performed in the hospital during the time embraced by the Report was about seven hundred, of which only four resulted fatally, and, adds Dr. Thompson, "During the last four years, not one single case of death has occurred as the result of surgical interference, although within that time a large number of very important operations have been performed, all of which have been successful."

We venture the assertion that such a remarkable degree of success as this statement indicates is wholly unprecedented in hospital practice.

From the summarized report we learn that there have been admitted to the hospital 11,455 patients, of whom 9,457 were cured, 1,081 relieved, 81 died, 257 were incurable, 7 were sent to Insane Asylum; in 442 the result of treatment was unknown, and 150 were still under treatment June 30, 1872.

The Report commences with the details of thirty-four operations for the radical cure of ruptured perineum, all of which were successful.

The operation for this accident as performed by Dr. Thompson does not differ in any important particular from that usually adopted by surgeons of the present day. It is essentially the same as that introduced by Dr. Baker Brown, differing from this latter, however, in that our author objects to the division of the sphincter ani except in special cases, preferring to rupture some of its muscular fibres by forcible dilatation, in the manner recommended and practiced by Professor Van Buren, of New York. He likewise prefers silver wire to the waxed thread both for the deep and superficial sutures; and instead of encouraging a constipated condition of the bowels after the operation, he endeavors to secure their daily evacuation.

Next follow the reports of two interesting cases of vesico-vaginal fistula, and in the chapter on this subject our author commits the blunder of introducing eight pages of unnecessary matter in the shape of an extract from Dr. Sims' address to the New York Academy of Medicine—a very excellent thing in its place, but very much out of place, we think, in this report. And inasmuch as we have very little fault to find either with our author or his book—this latter being for the most part excellent—it will be as well, perhaps, that we should put all our fault-finding in this one place. The introduction of the lengthy extract just alluded to is but one evidence of what seems to have been a governing principle with Dr. Thompson, namely, to make a very large book from very little material. Other examples of this disposition to fill up space are the introduction of Tyler Smith's description of the minute anatomy of the os and cervix uteri, which, with the woodcuts accompanying it, occupies nineteen pages; Morgagne's cases of foreign bodies in the bladder, thirteen and a half pages, and various authors on cancer, sixteen pages. The wood engravings—most of which, by the way, are wretched specimens of the art—are made to occupy much more space than necessary; and when we consider that in addition to all this expansion nearly two-thirds of each page is blank margin, itwill be readily understood that the kernel of this book might have been placed in a much smaller shell.

The remaining chapters of the book are devoted to the following subjects: Vaginal Cystocele and Rectocele; Diseases and Displacements of the Uterus; Uterine Tumors; Carcinoma Uteri; Diseases of the Vagina and Cervix Uteri; Chronic Cervical Metritis and Endometritis; Foreign Bodies in the Bladder; Extirpation of the Parotid Gland; Pelvic Cellulitis; Diseases of the Rectum. Details of cases under each head, the kind of operation employed, and the result of treatment, are fully given.

The Appendix consists of reports from the surgeons and physicians in charge of the different departments of the hospital dispensary, as follows: Diseases of Women, by Dr. F. A. Ashford; Diseases of Children, by Dr. Samuel C. Busey; Diseases of the Eye and Ear, by Dr. D. Webster Prentiss. These reports each contain much valuable matter, although their usefulness is somewhat marred, like that of the main report, by the too frequent evidences of hasty preparation. A. R. J.

*A Treatise on the Pneumatic Aspiration of Morbid Fluids: a Medico-Chirurgical Method of Diagnosis and Treatment of Cysts and Abscesses of the Liver, Strangulated Hernia, Retention of Urine, Pericarditis, Pleurisy, Hydrarthrosis, etc.* By GEORGES DIEULAFOY, Gold Medallist of the Hospitals of Paris. Philadelphia: J. B. Lippincott & Co. 1873.

A complete summary of the ideas which have occupied the medical mind concerning the injurious influence of fluid accumulations in closed cavities and organs of the body, the advantage to be derived from their removal, and the various appliances which have from time to time been adapted to the accomplishment of that object.

Whilst the disastrous effects of the accumulation of morbid fluids in cavities and organs have been recognized and appreciated from a very early period of the history of medicine, as is manifest from the numerous attempts which have been made to effect their removal, the true principle upon which such attempts must necessarily rest for their success has, it seems, eluded the perception of all experimenters, until the presentation of the essay of Dr. Dieulafoy to the French Academy of Medicine in 1867.

This true principle of pneumatic aspiration, as laid down by Dr. Dieulafoy, "is invariable; it consists in the fineness of the needle, and the creation of a previous vacuum;" in other words, upon the exclusion of air and the prevention of the escape of the fluids interstitially. The author's experiments have directed him to the following conclusion: "*It is always possible, owing to aspiration, to search for a fluid collection, without any danger, whatever may be its seat or nature.*" A proposition so bold and radical demands for its substantiation the most abundant and convincing evidence, inasmuch as its establishment must revolutionize many of our notions which time and prescript have rendered almost axiomatic. It is but just to the author, however, to admit that his numerous carefully conducted and conclusive experiments have supplied all the evidence which the most incredulous could demand, and mark the introduction of pneumatic aspiration as the beginning of a new era in rational medicine—constituting a new triumph of logical induction from observed phenomena to a scientific principle, entitled to take rank with vaccination and auscultation. H.

*Lectures on Madness, in its Medical, Legal and Social Aspects.* By EDGAR SHEPPARD, M.D., Member of the Royal College of Physicians, Professor of Psychological Medicine in King's College, London, and one of the Medical Superintendents of the Middlesex County Lunatic Asylum at Colney Hatch. Philadelphia: Lindsay & Blakiston. 1873.

The Council of King's College probably never demonstrated "their wisdom" more clearly than when "honoring an old student" with the appointment to the chair of Psychological Medicine in the institution under their charge. We have but one objection to the book, its brevity. What Dr. Sheppard has told us about insanity is so well told, so clear, so concise, so practical, that we close the book with regret that he has not gone more fully into detail and elaborated its various subjects more minutely; and yet that would have impaired the usefulness of the lectures for their destined end, *i. e.*, the instruction of students in the elements of psychological medicine; and for this end we know no work in the English language so well adapted. Dr. Sheppard gives in these lectures his own conclusion regarding insanity, which although by no means dogmatical, are independent, and, as far as we are able to judge, logical. This little book, while intended for the instruction of medical students, would conduce greatly to the enlightenment of lawyers and judges, and tend to remove from their minds the influence of certain legal hypotheses regarding insanity, which, based upon false physiology, have served as foundations for prejudice and injustice. When these begin to study madness from a physiological rather than from a metaphysical standpoint, then will we begin to correctly estimate its social and legal relations, and definitely to fix the status of its victims. H.

*Insanity and its Relations to Crime. A Text and a Commentary.* By WM. A. HAMMOND, M.D., Professor of Diseases of the Mind and Nervous System, and of Clinical Medicine in the Bellevue Hospital Medical College, etc., etc., etc.

Upon the vexed question, which the title to the book before us involves, law and medicine seem to be arrayed on opposite sides; the first claiming as its victims all who transgress its edicts, no matter what their mental condition, provided "they can distinguish right from wrong;" the second claiming as its patients those whose transgressions are involuntary. The reader of Dr. Hammond's "Text and Commentary," will entertain a reasonable doubt whether the author may not have "gone over to the other side," and assumed the role of counsel for the prosecution, endeavoring to secure the (almost) indiscriminate punishment of those whose protection he should be most desirous to secure. We should, however, be sorry to believe that the majority of the legal profession endorse some of the propositions advanced by the author, in which he appears to separate the rights of society from those of the individual, and to establish an antagonism between them which appears to be totally illogical. The proposition that "society has no more business with abstract justice than it has with any other bit of philosophy," savors too strongly of Communism.

We cannot but think that in his zeal for the protection of society and the punishment of crime, the author disregards too blindly the protection of individuals, the social units, without which society would not exist.

The consequences which might be logically deduced by extension of the parallel cited below, would be to reduce society to a condition horrible to contemplate. "Even if actual cerebral disease be the cause of the irresistible impulse, it does not materially detract from the right of society to protect itself against injury from those in whom it exists." To this there can be no objection, but mark the mode which is suggested in the context, viz.: "A dog afflicted with hydrophobia going about snapping at those who come in its way, is destroyed in order that it may not bite us, and may not poison other dogs, who, in their turn, may bite us. The dog is suffering from a disease of its brain which gives it an irresistible impulse to bite. We kill it without the slightest hesitation." We would be almost equally justifiable in killing the insane with irresistible impulses to commit homicide, if we did not possess places in which to confine them safely. An extension of such reasoning would justify the killing of a small-pox patient in order to protect ourselves from the probable fatal consequences of contagion.

If the fallacy of such reasoning needed demonstration it would be found in the spirit of Christian philanthropy of the peasants of Gheel, in Belgium, where every cottage is a lunatic asylum and every peasant a keeper. H.

*Pharmaceutical Lexicon: a Dictionary of Pharmaceutical Science.* Containing a Concise Explanation of the Varied Subjects and Terms of Pharmacy, with Appropriate Selections from the Collateral Sciences, etc., etc. By H. V. SWERINGEN, Member of the American Pharmaceutical Association, etc. Philadelphia: Lindsay & Blakiston. 1873.

Inasmuch as the author of the above work, in his preface, deprecates criticism, we forbear to say much that might be said of the injurious influence of compendiums in general, upon the progress of scientific study.

Such a work as this, while doubtless written in good faith, to subserve the purpose of a work of reference for the busy pharmacist or physician, is liable to the abuse of encouraging idleness and ignorance, by giving to the pretender and sciolist a "ready method" of seeming to possess knowledge only to be truly acquired by long years of patient study and investigation.

We must protest against the author's philological theories advanced in the preface and practically applied throughout the book. Words, independent of their derivations, are arbitrary symbols, empty sounds, whose use simply hastens the process of phonetic corruption which all languages seem doomed to sustain, and from which it should be the duty of every scholar to protect them.

The Pharmaceutical Lexicon certainly contains a good deal of useful information, and thus far we commend it cheerfully to those who need a manual of quick and easy reference for popular use. H.

*Contributions to Practical Surgery.* By GEORGE W. NORRIS, M.D., late Surgeon to the Pennsylvania Hospital; Vice-President of the College of Physicians of Philadelphia, etc., etc. Philadelphia: Lindsay & Blakiston. 1873.

The above is a republication in book form of the author's essays which have already appeared from time to time in the *American Journal of Medical Sciences*, and present his views and opinions upon the various points in practical surgery which they comprehend. The opinions of Dr. Norris are entitled to respect, not only from his well-earned reputation as a surgeon, and his long experience and ample opportunities for observation and experiment, but also from the mass of evidence brought to sustain them in the shape of statistics carefully tabulated and apparently honestly compiled.

That the statistics do not include operations of very recent date, is to be regretted, as they might thus escape the suspicion of unfairness, to which most statistical arguments are susceptible. They will, however, be very acceptable to a numerous class of practitioners dependent upon the opinions of standard authorities, and not having sufficient data within their own experience to justify the formation of individual conclusion. The whole book bears the impress of the patient and laborious study, the accurate analysis, and the conscientious criticism of a skillful surgeon, and an accomplished scholar. These "Contributions to Practical Surgery" will be welcomed by the profession and accepted as actual additions to the stock of human knowledge. H.

